# Supplementary material for: Large-Size Subunit Catalases Are Chimeric Proteins: A H2O2 Selecting Domain with Catalase Activity Fused to a Hsp31-Derived Domain Conferring Protein Stability and Chaperone Activity
Source: Antioxidants (Basel). 2022 May 17;11(5):979. doi: 10.3390/antiox11050979 (PMC9137513; doi:10.3390/antiox11050979)
Supplement: Supplementary file 1 [file antioxidants-11-00979-s001.zip › antioxidants-1698224-supplementary-final/Table S2 Figures S1-S4.pdf]

Table S2. Frequency (%) of amino acid in the catalytic and the chaperone domain of LSCs

AA	H_r[a]	DP[b]	PDB[c] ±20%		CATw[d]		CAT_{HC}[e]		$L1_{C-T}$[f]		$L2_{C-T}$[f]		Bac_{C-T}[g] .	
I	0.00	- 0.33	5.91 (4.7-7.1)	- 1.95	**4.17**	- 1.39	4.34	- 1.45	6.25	- 2.08	**2.80**	**- 0.93**	**4.38**	**- 1.46**
V	0.46	- 0.29	6.86 (5.5-8.2)	- 1.99	6.50	- 1.89	6.21	- 1.80	**8.90**	- 2.58	**11.15**	- 3.23	**8.98**	**- 2.60**
A	0.47	- 0.04	8.25 (6.6-9.9)	- 0.33	7.91	- 0.32	6.64	- 0.27	**13.55**	- 0.54	**13.35**	- 0.53	**17.70**	**- 0.71**
L	0.79	- 0.20	9.65 (7.7-11.6)	- 1.93	**6.76**	- 1.35	**6.75**	- 1.35	**6.15**	- 1.23	8.70	- 1.74	9.57	- 1.91
G	0.80	- 0.12	7.07 (5.7-8.5)	- 0.85	7.61	- 0.91	7.42	- 0.89	**9.15**	- 1.10	**11.45**	**- 1.37**	**11.26**	**- 1.35**
M	0.38	- 0.05	2.41 (1.9-2.9)	- 0.12	**1.87**	**- 0.09**	**1.89**	**- 0.09**	**1.55**	**- 0.08**	**0.45**	**- 0.02**	**1.40**	**- 0.07**
C	0.59	- 0.31	1.38 (1.1-1.7)	- 0.43	**0.46**	- 0.14	**0.50**	- 0.16	**0.30**	**- 0.09**	**0.00**	**- 0**	1.05	- 0.32
			41.53 (33-50)	- 7.60	35.28	- 6.09	33.75	- 6.01	45.90	- 7.70	47.90	- 7.82	53.62	- 8.42
					- 15.05%		-18.73%		+10.5%		+15.3%		**+29.11%**	
T	0.66	0.00	5.35 (4.3-6.4)		5.56		6.18		5.35		6.10		3.61	
S	1.46	+0.33	6.64 (5.3-8.0)	+2.21	5.29	+1.76	5.12	+1.71	7.10	+2.37	7.60	+2.53	4.56	+1.52
N	2.03	- 0.20	4.06 (3.2-4.9)	+0.81	**5.23**	+1.05	4.89	+0.98	**2.40**	**+0.48**	**2.25**	**+0.45**	**1.22**	**+0.24**
Q	1.26	+0.46	3.93 (3.1-4.7)	+1.81	4.40	+2.02	4.31	+1.98	**2.70**	**+1.24**	**3.15**	**+1.45**	**1.78**	**+0.82**
R	1.60	+0.09	5.53 (4.4-6.6)	+0.50	5.99	+0.54	5.73	+0.52	5.25	+0.46	**3.55**	**+0.32**	5.16	+0.46
K	2.76	+0.40	5.80 (4.6-7.0)	+2.32	4.93	+1.97	4.87	+1.95	5.30	+2.12	5.30	+2.12	**3.83**	**+1.53**
D	3.20	- 0.03	5.46 (4.4-6.6)	+0.16	**6.81**	+0.20	**6.98**	+0.21	**4.65**	+0.14	**7.30**	**+0.22**	**7.49**	**+0.22**
E	3.32	+0.66	6.72 (5.4-8.1)	+4.42	5.61	+3.70	5.61	+3.70	6.10	+4.03	**5.30**	**+3.50**	**4.82**	**+3.18**
			43.49 (34-52)	+12.23	43.82	+11.24	43.69	+11.05	38.65	+10.84	40.55	+10.59	32.47	+7.97
									-11.13%				**-25.34%**	
H	2.29	- 0.10	2.27 (1.8-2.7)	- 0.23	**3.55**	**- 0.36**	**3.94**	- 0.39	**3.25**	- 0.33	**0.15**	**- 0.02**	2.63	- 0.26
P	0.55	+0.57	4.73 (3.8-5.7)	+2.70	**6.41**	**+3.65**	**6.68**	+3.81	3.65	+2.08	**3.20**	**+1.82**	4.69	+2.67
F	0.24	- 0.33	3.86 (3.1-4.6)	- 1.29	**5.97**	**- 1.99**	**7.10**	- 2.37	**4.70**	- 1.57	**5.20**	**- 1.71**	3.36	- 1.56
W	0.65	- 0.60	1.10 (0.9-1.3)	- 0.66	**1.44**	**- 0.86**	**2.00**	- 1.20	1.00	- 0.60	**0.50**	**- 0.30**	1.00	- 0.60
Y	0.83	- 0.44	2.92 (2.3-3.5)	- 1.28	3.36	- 1.48	3.12	- 1.37	2.90	- 1.28	2.45	- 1.08	**1.37**	**- 0.60**
			14.88 (12-18)	- 0.76	20.73	- 1.04	22.84	- 1.52	15.50	- 1.70	11.50	- 1.29	13.06	- 0.35
					**+39.3%**		**+53.5%**				**-22.72%**		-12.25%	
Instability factor				+3.87		+4.11		+ 3.52		+1.44		+1.48		- 0.8

Table S2. Frequency (%) of amino acids in the catalytic and the chaperone domain of LSCs. Frequency of amino acid residues that are 20% higher or more than of the PDB mean are shown in black bold letters; frequency that are 20% lower or more are shown in gray bold letters. AA, the 20 amino acids, ordered as hydrophobic, hydrophilic, or heterocyclic/aromatic. [a.]Hydrophobicity scale for amino acid residues determined at the protein surface; scale is derived from the principal component region but has some overlap with other regions; amino acid hydrophobicity of some residues varies according to the hydrophobic or hydrophilic surrounding [1]. [b] Disorder propensity (DP) taken from [2, figure 6]. [c] SwissProt Database statistics mean frequency of amino acids; in parenthesis are values that are ±20% of the mean. [d] Mean amino acid frequency of 38 different catalases cut at the first conserved residue of the N-terminal end and, in the case of the 7 LSCs of the same list, the C-terminal domain was eliminated. [e] Mean frequency values of 38 different catalases (Table S1) cut at the first conserved residue at the N-terminal end and at the middle of the wrapping loop, which is the sequence that defines the heme pocket. [f] Mean frequency value of the first 19 orthologues each in a Blast search with CAT-1 and CAT3. [g] C-terminal domain mean value of the 19 orthologous of *Cereibacter sphaeroides katE* and other the different groups of bacterial LSCs (Table S1).

1) Di Rienzo L, Miotto M, Bò L, Ruocco G, Raimondo D and Milanetti E (2021) Characterizing Hydropathy of Amino Acid Side Chain in a Protein Environment by Investigating the Structural Changes of Water Molecules Network. Front. Mol. Biosci. 8:626837. doi:10.3389/fmolb.2021.626837

2) Shimizu, K.; Muraoka, Y.; Hirose, S.; Tomii, K.; Noguchi, T. Predicting mostly disordered proteins by using structure-unknown protein data. BMC Bioinformatics 2007, 8, doi:10.1186/1471-2105-8-78.

CAT-1 treated with Subtilisin looses its CT but remains active

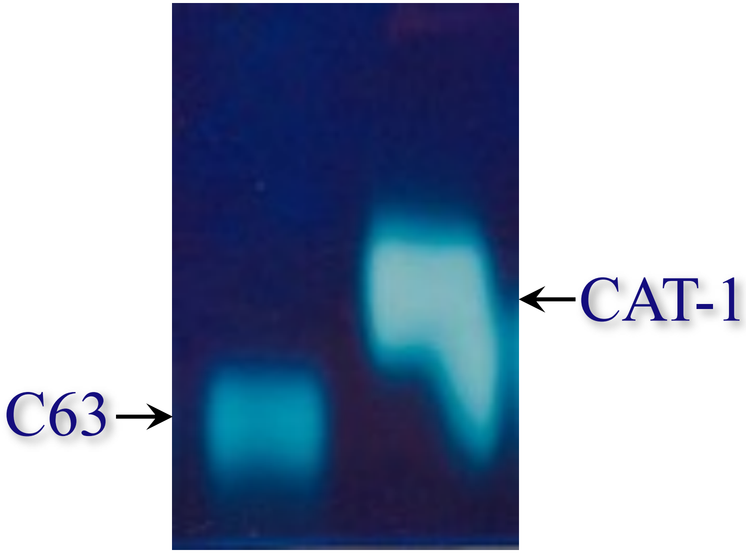

PAGE: non-denaturing conditions, stained for catalase activity

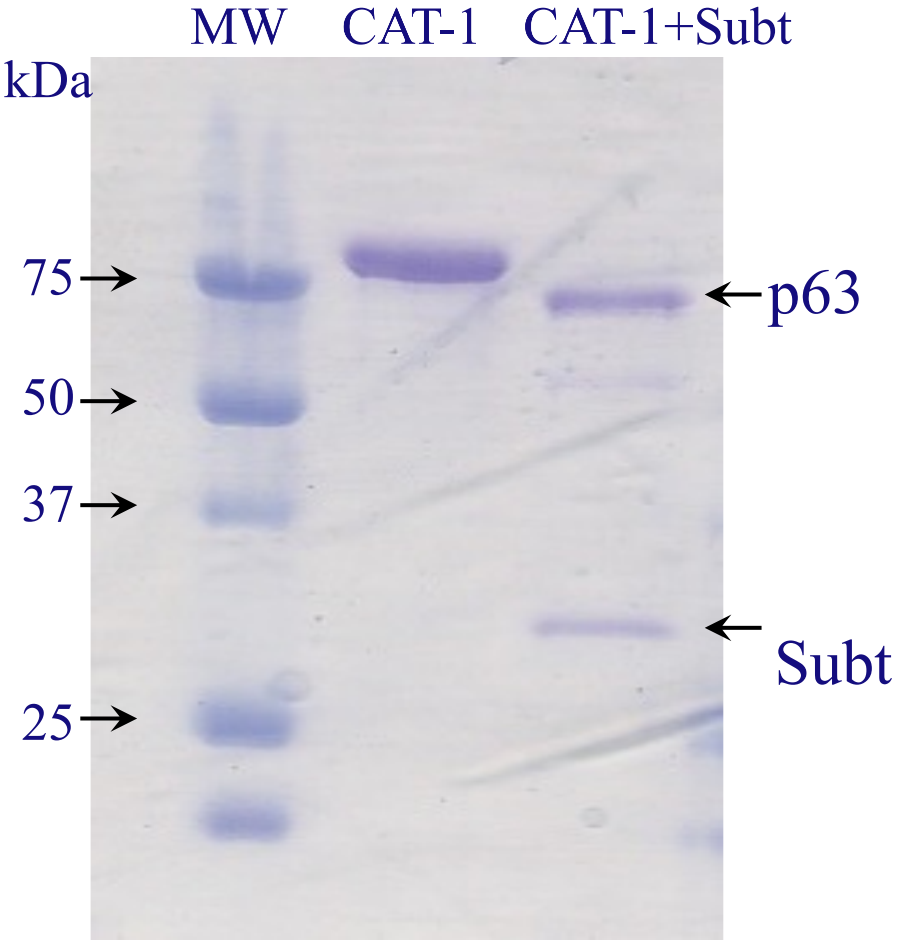

PAGE: denaturing conditions, stained for proteins

C63 = CAT-1 without its CT domain; p63 = 63 kDa peptides
Subt = Subtilisin

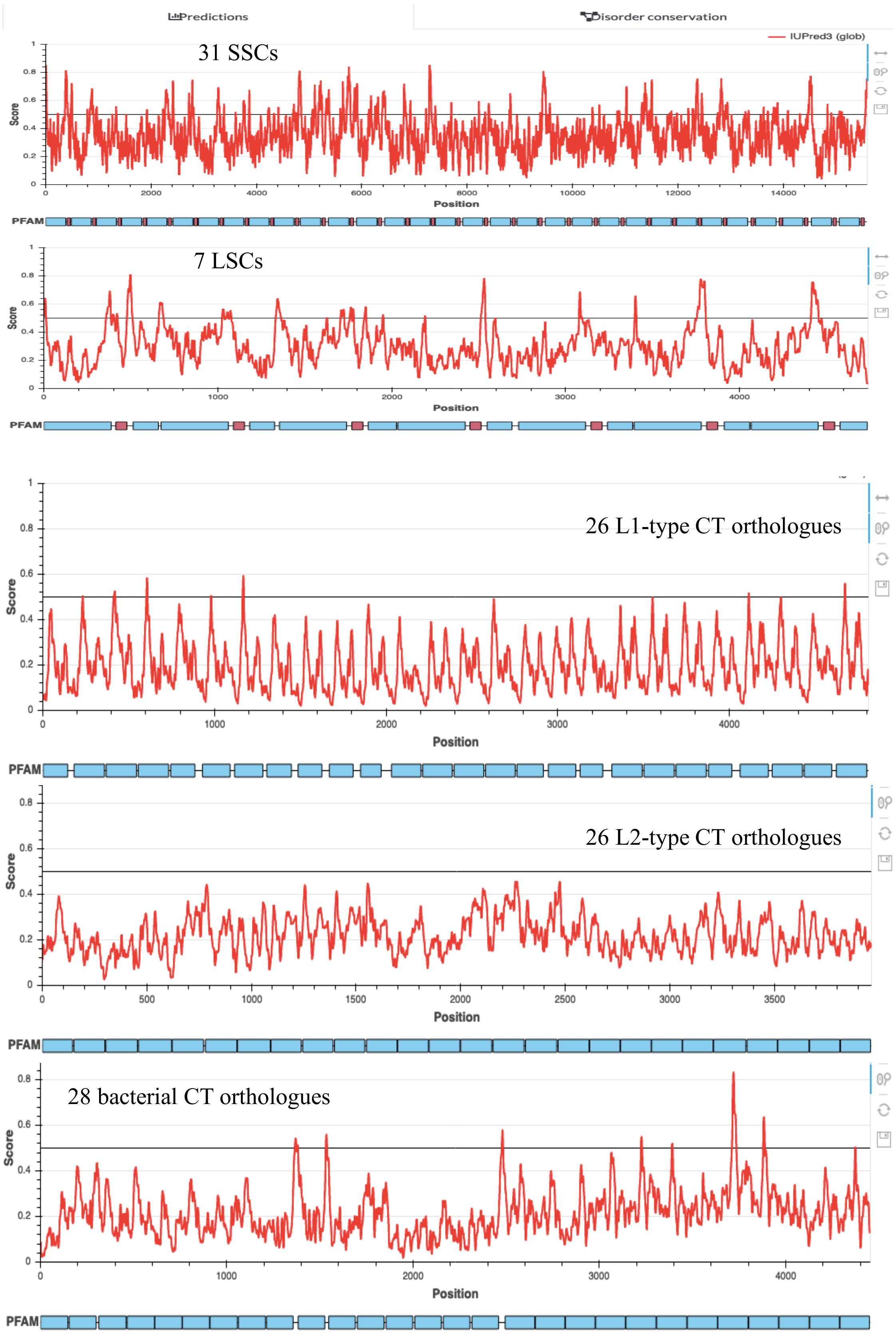

Figure S2. Stability prediction of SSCs (31), LSCs (7) and CT domains of fungal L1-type (26), L2-tye (26) and bacterial (28). LSCs are more stable than SSCs and CT domains are very stable.

```
YHBO_ECOLI              ----------------------------------------------------------------MSKKIAVLITD
Hsp31_Neu_cra           ---------------------------------------------------------------MSAPKVLVVLTS
ProtI_Amy_met           -----------------------------------------------------------MANTLSGKRVAFVVAP
L2_Pen_bra_MC_CT        -----------EVPEPDPTFYHDNT----------TANIGAFGQKLL--KLDGL-------------KV-GVLAS38
L1_Aur_mel_MC_CT        -----------DIPEEARRV---NTG------KRSKN-LSQE--DFPPKNLTIA-----------TRMVAILIAD41
HCHA_ECOLI              MTVQTSKNPQVDIAEDNAFFPSEY------------S-LSQY--TSPVSDLDGVDYPKPY-RG-KHKIL-VIAAD57
Bac_MC_CT_Kib_phy       --------------------PAPAG-EPANDVTTSPA-LSQVV-TEPG---PIA-----------GRKI-GVIAD37
MC_CT_Str_alb           --------------------PAPAAGEPLADPSPSPA-LSQVGQTWPPD----------------GRIV-GIVAD37
YajL_ECOLI              ----------------------------------------------------------------MSASALVCLAP
DJ1_Tul_sp              -----------------------------------------------------------------MPSALILVAN
Intr_Prot_Des_sp        ------------------------------------------------------------------MKALILSAD
```

Figure S3 A. The mobile coil of LSCs has similarity with the N-terminal sequence of bacterial Hsp31 (HchA). Alignment of one representative protein sequence of YhbO, fungal Hsp31, Protease I, YajL, fungal DJ-1 and Intracellular protease together with the MC_CT of bacterial and fungal (L1, L2) LSCs. The mobile coil of the four groups of LSCs has sequence similarity with the N-terminal domain of bacterial Hsp31 (HchA). The other DJ-1/PfpI proteins and the fungal Hsp31 do not show sequence similarity with this region.

```
L2_Pen_bra_MC_CT       76NQTYSA-TDAINFDAVIVADGAEGLFGSNSFTASPT-AKASGATSL-YPAGRPLEILVDAFRFGKPVGTLGQGVLALDD149
Bac_MC_CT_Kib_phy      73ERTLLT-ARSIEFDAVVVAGGTA-----------------------PTNDIKLVLLLQEAFRHCKAIAAWGTGQDILED134
MC_CT_Str_alb          79QRTFAT-ARSVEFDAVMLAGVPARG--ADALGSRD--AKAGDAQAAAATDPRLLLLVSEAYRHGKAIGAWNSGEQVLTA152
L1_Aur_mel_MC_CT       96DHNLEG-QRSTMYDAIFIPGGADSI-------------------ATLRKNGRALHYVREAFGHLKSIGATGEAVAFVKD146
Hsp31_Neu_cra          90E-FVGNPSAIAPYSAIFFPGGH-GPM------------------YDLATSSESQQLIREFWDAGKTVAAVCHGPAALVN149
HCHA_ECOLI            135D-VVASLNADSEYAAIFVPGGH-GAL------------------IGLPESQDVAAALQWAIKNDRFVISLCHGPAAFLA193
DJ1_Tul_sp             60DIDLRDLEKPREYDALIIPGGAQGA-------------------ETLSGSSVVQQLVQDYYKSGKLVGMICAGSLVALR120
YajL_ECOLI             55DAPLVEVA-DGEYDVIVLPGGIKGA-------------------ECFRDSTLLVETVKQFHRSGRIVAAICAAPATVLV114
Intrac_Prot_Des_sp     50DKTIDEV-NPDDYAILILPGGK-AP-------------------VEVRKDPKALEIARSFFAGSKPVTAICHGPQILIS108
ProtI_Amy_met          62DKVVTDV-SPDDFDALVLPGGVANP-------------------DFLRTVPDAVRFVRGFFSQQKPVAAICHAPWTLIE121
YHBO_ECOLI             54DKSIDEV-TPAEFDALLLPGGH-SP-------------------DYLRGDNRFVTFTRDFVNSGKPVFAICHGPQLLIS112
                         :           :  :..  .                                       :    : :     .   :
AraC_Pec_car            8ESQPNPLLPGYSFNAYLVAGL28                 69FFCNPG74       292AAKTLPGTWGERILNVIS309
                                                                     215RLAHLFREQV224
```

Figure S3 B. Proteins of the DJ-1/PfpI superfamily and the CT of LSCs show sequence similarity in two contiguous hydrophobic amino acid sequences flanked by charged amino acid residues. Alignment of one representative protein sequence of the DJ-1/PfpI superfamily: YhbO, fungal Hsp31, Protease I, YajL, fungal DJ-1 and Intracellular protease and the MC_CT of representative sequences of four groups of LSCs. AraC shows sequence similarity with this region with non-contiguous stretches of hydrophobic residues and charged amino acid residues.

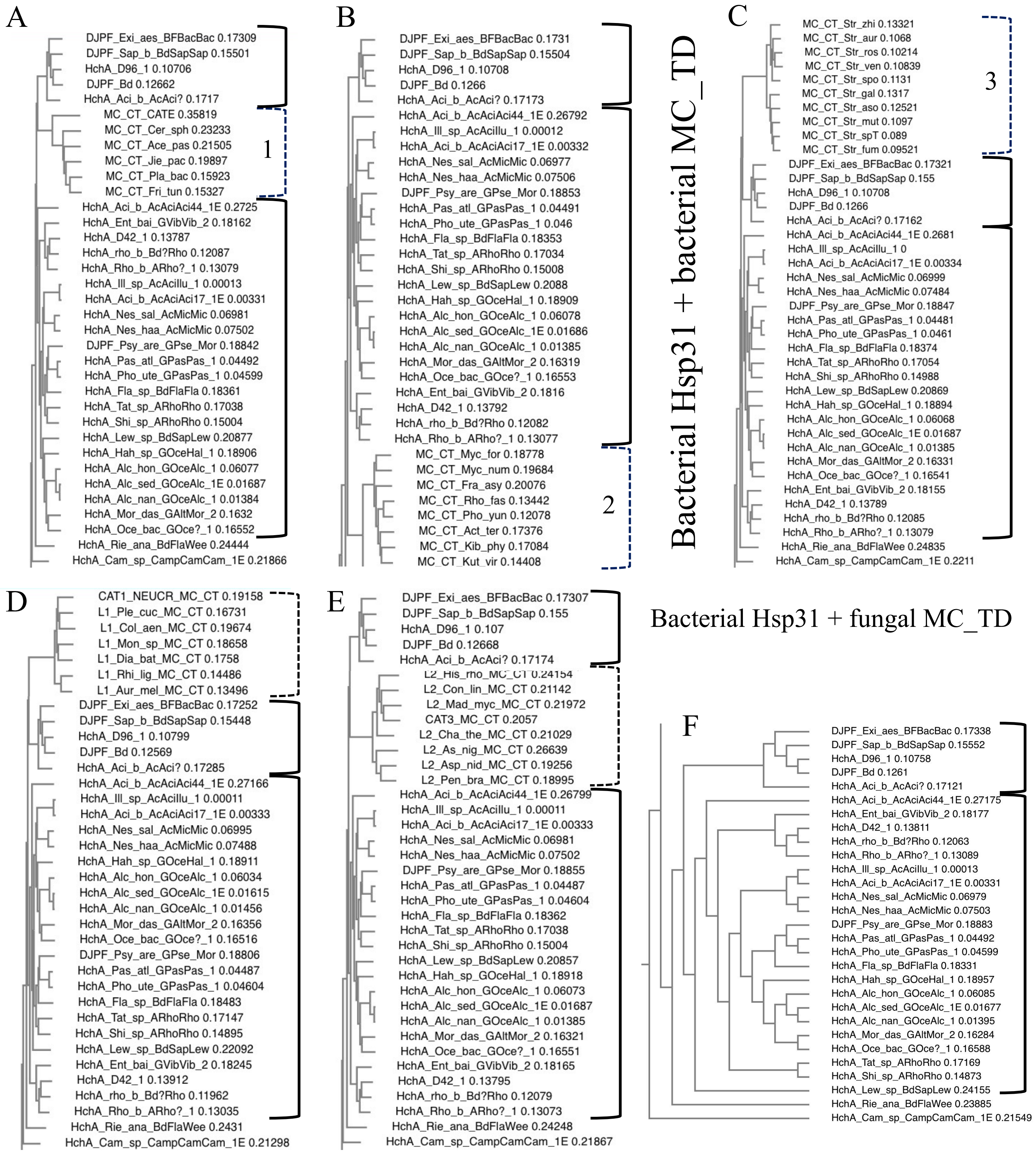

Figure S4. Phylogeny of bacterial Hsp31 and the MC_TD of representative bacterial and fungal LSCs. HchA sequences (186) from different phyla and families of bacteria were aligned together with the MC_TD of three groups of bacterial and two of fungal LSCs. (**A**) With MC_TD of bacterial group-1; (**B**) With MC_TD of bacterial group-2; (**C**) With MC_TD of bacterial group-3 (Streptomyces); (**D**) With MC_TD of fungal L1-group; E) With MC_TD of fungal L2-group; (**F**) Cladogram of the two groups of Hsp31 sequences that are closest to all MC_TD. A group of 5 sequences of Hsp31 align preferentially with group-1 and group-3 of bacterial and both groups of fungal MC_TD and includes: one Bacillota (Firmicutes) (Exiguobacterium aestuarii), one Actinomycetota (Acidimicrobiia) two Bacteroidota and one Pseudomonadota delta. Other 22 sequences are also consistently related to all MC_CTs.
